# AI approaches for the discovery and validation of drug targets

**DOI:** 10.1017/pcm.2024.4

**Published:** 2024-05-24

**Authors:** Aaron Wenteler, Claudia P. Cabrera, Wei Wei, Victor Neduva, Michael R. Barnes

**Affiliations:** 1 Digital Environment Research Institute, Queen Mary University of London, London, United Kingdom; 2 Centre for Translational Bioinformatics, William Harvey Research Institute, Queen Mary University of London, London, United Kingdom; 3 MSD Discovery Centre, London, United Kingdom; 4 NIHR Barts Cardiovascular Biomedical Research Centre, Barts and The London School of Medicine and Dentistry, Queen Mary University of London, London, United Kingdom; 5 The Alan Turing Institute, London, United Kingdom

**Keywords:** drug discovery, drug targets, artificial intelligence, machine learning, multiomics

## Abstract

Artificial intelligence (AI) holds immense promise for accelerating and improving all aspects of drug discovery, not least target discovery and validation. By integrating a diverse range of biological data modalities, AI enables the accurate prediction of drug target properties, ultimately illuminating biological mechanisms of disease and guiding drug discovery strategies. Despite the indisputable potential of AI in drug target discovery, there are many challenges and obstacles yet to be overcome, including dealing with data biases, model interpretability and generalisability, and the validation of predicted drug targets, to name a few. By exploring recent advancements in AI, this review showcases current applications of AI for drug target discovery and offers perspectives on the future of AI for the discovery and validation of drug targets, paving the way for the generation of novel and safer pharmaceuticals.

## Graphical abstract

**Figure:**
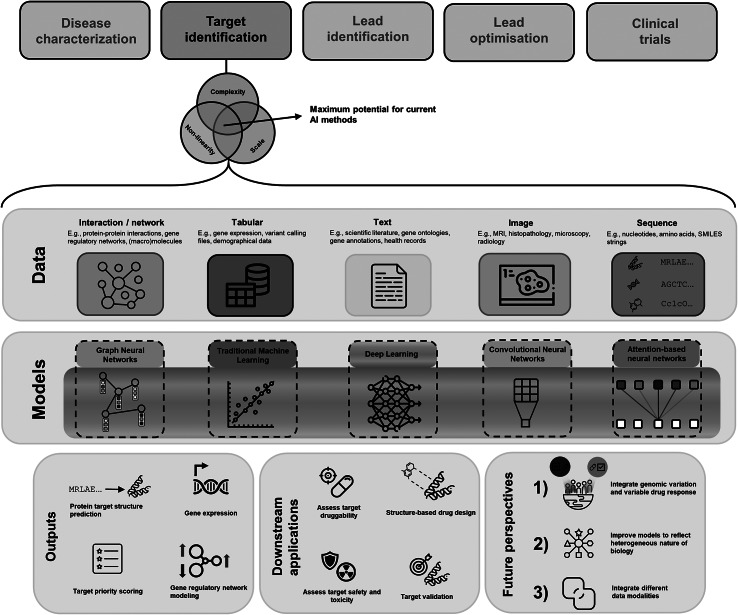

## Impact statement

Artificial intelligence (AI) is transforming drug discovery and development by enabling the rapid analysis of massive amounts of biological data and chemical information. This paper reviews recent advances in using AI methods for the discovery and validation of drug targets. Identifying and validating novel drug targets is fundamental to creating safe and effective new medicines but has remained a major bottleneck in the drug R&D process. By integrating diverse datasets, AI models can accurately predict key properties of drug targets, reveal intricate biological relationships underlying disease, and guide drug discovery strategies. This paper highlights groundbreaking applications of AI that accelerate target discovery, including models that prioritise candidate genes, predict druggability of proteins, uncover disease mechanisms, and simulate biological experiments. Critically, AI enables leveraging insights across modalities like sequences (e.g., DNA, proteins), structures (e.g., compounds, proteins), multiomics, biomedical literature and more. Integrating multimodal inputs is paramount for comprehensively understanding complex diseases involving genetic and non-genetic factors. The AI methods outlined will profoundly enhance R&D efficiency. By illuminating novel drug targets, AI-powered target discovery will expand treatment options available for patients suffering from previously untreatable or poorly managed diseases. From rare diseases and refractory cancers to multifactorial neurodegenerative and autoimmune conditions, accelerating target discovery through AI has far-reaching therapeutic implications. Additionally, safer, more selective drugs developed against AI-predicted targets could dramatically improve patient outcomes and quality of life. Overcoming existing challenges in AI-based target discovery will be critical to actualising its immense potential and promises to usher in a new era of data-driven, accelerated drug R&D.

## Background

Historically, drug target discovery and validation has been a laborious and somewhat haphazard process, heavily reliant on industry standard laboratory models and analysis procedures (Drews, 2000; Huang et al., 2004; Materi and Wishart, 2007). Most drug discoveries to date have taken a phenotype-first approach focusing on the evaluation of the therapeutic potential of compounds in phenotypic assays, without necessarily knowing the exact target or mechanism of action (Moffat et al., 2017). This approach relies largely on serendipity, where complex compound libraries, including phytochemicals, biochemicals and other organic chemistry, are identified for therapeutic use by chance. Naturally, pharma companies initially sought to improve their odds by increasing the size and complexity of their compound libraries, and by the mid-2000s most major pharmaceutical companies had compound libraries in the range of 1–2 million small molecule entities (SMEs) (Hann and Oprea, 2004). However, the unsustainability of this chemistry arms race has spurred a shift towards a target-first strategy, which signified a pivotal moment in pharmacological research, emphasising the importance of thorough understanding and validation of a biological target before initiation of the drug design process. This paradigm shift marked a transition from empirical, trial-and-error methods to a more rational and systematic approach, greatly enhancing the efficiency and effectiveness of drug discovery. Ironically, although the target-first approach was designed to reduce the complexity of drug discovery, it may have had the opposite effect, simply highlighting the challenges of true target validation, leading to over a decade of increased failure in drug discovery stemming from poorly validated targets (Paul et al., 2010; Scannell et al., 2012). With an increasing repertoire of biomolecular assays to probe mechanisms such as CRISPR-Cas9, so-called target deconvolution studies have been conducted. These studies connect phenotypic to target-first approaches by attempting to elucidate the mechanism of action of the target upon which a drug acted retrospectively. This strategy enriches the phenotype-centric drug discovery paradigm with mechanistic understanding of the observed therapeutic effect and sets the groundwork for integration of phenotype-first and target-first approaches (Terstappen et al., 2007).

In this review, we define drug targets as biomolecules—primarily proteins, but also DNA, RNA or other biomolecular species—that a therapeutic compound can bind to or modulate. The pool of existing drug targets is limited, and assessments of the druggable genome, which refers to those genes susceptible to modulation by small molecules, fluctuate. The latest estimate places this number at 4,479 potential targets, accounting for approximately 22% of protein-coding genes (Finan et al., 2017). According to records of the Human Protein Atlas (HPA), there are approximately 863 FDA approved drug targets (Paananen and Fortino, 2020), over 50% of these targets are represented by just four protein families—ion channels, nuclear receptors, kinases, and G-protein coupled receptors (Bakheet and Doig, 2009; Santos et al., 2017). When it comes to finding novel, efficacious, and safer drug targets, as a general guideline, targets should have a role in disease, limited role in normal physiology, particularly in critical tissues such as the heart, and ideally should be druggable with small molecules, although biologic drugs and gene targeted therapies make almost all targets therapeutically tractable. Furthermore, while a laboratory-resolved 3D protein structure was a prior requirement for drug design, with the advent of protein structure prediction models, further accelerated by AI approaches (Baek et al., 2021; Jumper et al., 2021; Lin et al., 2023b), high-quality 3D structures of a wide range of potential drug targets are generally available. This enables a broader application of *in silico* structure-based drug design. Another desirable property for a drug target is having multiple binding pockets. By having multiple potential binding pockets, different conformations of the protein in various functional states can be targeted. It also provides opportunities for identifying allosteric inhibitors rather than only targeting the active site. Allosteric sites may offer better selectivity and provide safety benefits (May et al., 2007; Abdel-Magid, 2015; Wagner et al., 2016b). Lastly, by understanding the associated pathways of the target, we gain insight into the processes the target is involved in and thus, what other biological processes could potentially be affected. This can help the assessment of potential off-target effects.

Despite the great progress in drug discovery, the process is still burdened by high costs, long timelines, and extraordinarily high attrition rates in clinical trials, attributed to limited efficacy, safety concerns, off-target effects, or sometimes purely economic reasons (DiMasi et al., 2016; Minikel et al., 2024) Collectively, against this backdrop of failure, the need for transformative solutions for drug discovery becomes clear, especially when we consider our incomplete understanding of target mechanism and the vast chemical space of compounds that can interact with that target.

## The role of AI in drug discovery

Ideally, we would develop a comprehensive mathematical framework to systematically navigate the vast search spaces and intricate interactions inherent to drug discovery. However, realising such a framework has proven to be an immensely challenging endeavour with limited success so far. In contrast, methods using artificial intelligence (AI) are particularly well-suited for modelling the complexities and nuances of drug discovery. When employing AI, we essentially shift our approach: rather than relying on explicit mathematical descriptions of the underlying biology, chemistry, and physics, we leverage AI models to learn and infer patterns directly from data. While adopting data-driven machine learning techniques holds great promise for enhancing drug discovery pipelines, there are also certain trade-offs, such as a lack of transparency in the models and obscured understanding of causality.

AI has the potential to accelerate drug discovery by improving the identification of drug candidates and enhancing our understanding of their mechanisms. The increasing volume of diverse biological and chemical data, including genomics, proteomics, metabolomics, electronic health records, and biomedical literature, combined with high-throughput experiments, greatly enhances AI’s ability to extract and interpret insights. Notably, recent studies have highlighted the importance of including genetic and genomic data in drug target discovery pipelines (Razuvayevskaya et al., 2023). One estimate quantifying the impact genetic evidence has on the success of clinical trials estimated the odds of advancing to a later stage of clinical trials to be 80% higher when genetic evidence for a target is present (Minikel et al., 2024). Furthermore, AI can be used to develop *in silico* methods to predict and simulate biological and chemical spaces. Examples of such approaches are cellular and genetic perturbation modelling (Prasad et al., 2022; Bunne et al., 2023), gene expression prediction (Kelley et al., 2018; Avsec et al., 2021; Linder et al., 2023), variant effect prediction (Frazer et al., 2021; Brandes et al., 2022; Cheng et al., 2023; Lin et al., 2023a), protein structure prediction (Baek et al., 2021; Jumper et al., 2021; Lin et al., 2023b), drug-target interaction prediction (Chen et al., 2016; Wen et al., 2017; Huang et al., 2021), and molecular docking simulations for drug design (Gentile et al., 2020; Corso et al., 2023).

**Figure 1.**
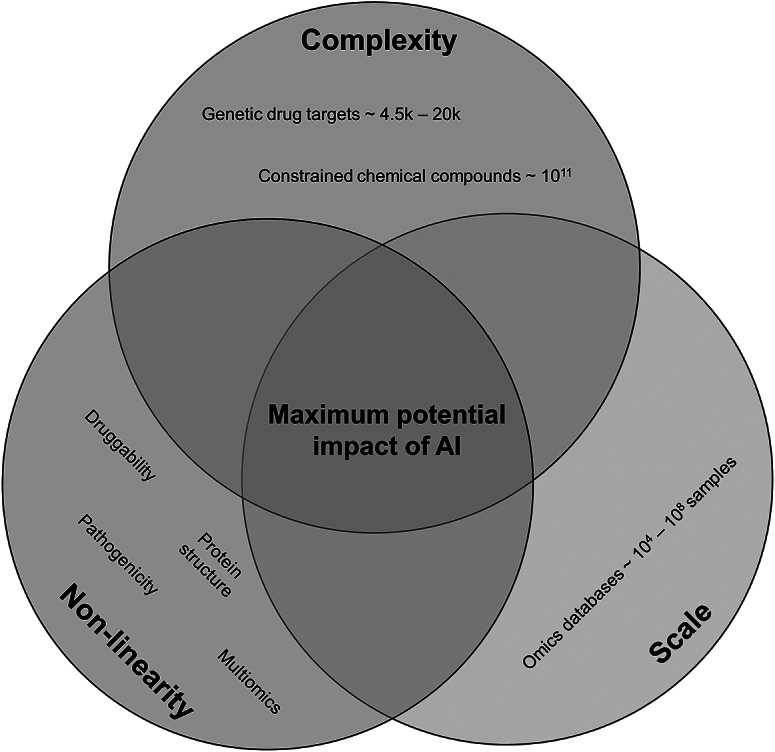
Venn diagram of guiding criteria for the maximum impact of AI in relation to drug discovery. We have made the connection to drug target discovery in the respective sets. The intersection of all sets is where the sweet spot for using AI lies.

When it comes to determining the applicability of AI, we can refer to some guiding principles (Figure 1) that can help us to establish whether introducing AI to solve our problem is sensible. We argue that drug target discovery problems lie at the intersection of all these principles, making them amenable to be solved with AI.

First, the problem at hand must have sufficient scale. Building a successful AI model is reliant on having examples to learn from. While unsupervised approaches can be powerful, the potential of AI predominantly resides in the ability to uncover generalisable patterns within training data through a supervised or a self-supervised framework. A part of this scale is the quality of the data. The dataset should not just be large, but it should also be of high quality or be processed such that it is of high quality. High-quality data implies that the model can learn meaningful signals from the patterns and relationships contained within the data. Some concrete examples of factors potentially decreasing data quality are noise, class imbalances, population bias, and missing data.

Second, the complexity of the problem should be appropriate to fully leverage the power of AI models. At the lower bound of the complexity spectrum, the problem could be insufficiently complex, making it likely that an overparameterized AI model is developed that performs seemingly well, but does not generalise. This phenomenon is referred to as overfitting in AI literature. Note that overfitting is not limited to this scenario and can also occur in poorly designed AI models where the problem itself is not necessarily insufficiently complex. At the other end of the complexity spectrum, a problem could be intractable. Take the entire chemical space of ~10^60^ compounds (e.g., Reymond, 2015), this immense search space is simply too large for any computational method to fully explore. However, we can make this task more manageable by focusing on a smaller, more relevant subset of compounds. One effective approach to achieve this is by using generative AI models. These models are trained by adding random variations to existing, known data and then attempting to reconstruct the original input from this altered data. Through this process, the model learns the patterns and distributions inherent in the data, which can be used to construct outputs based on these patterns.

In the context of drug discovery, this technique can be applied to known chemical structures. This is the basis of generative molecular design (GMD), where AI models are used to generate potentially viable chemical compounds by learning from existing chemical structures (Thomas et al., 2023). This approach helps streamline the search for new drug candidates by focusing on the most promising areas of the vast chemical space, in this case, up to ~10^11^ compounds (Ruddigkeit et al., 2012), constraining the search space and thus making the problem computationally tractable. For AI methods to thrive, a balance must be struck as it pertains to the complexity of the problem. We argue that drug discovery, including drug target discovery, satisfies the complexity criterion. Target discovery is often constrained to parameterisations of the genome or the druggable genome. These are about 20,000 and 4,000 genes in size, respectively, which is a tractable search space. As for the chemistry of compounds binding to the target, we can narrow down the search space to effectively design novel compounds.

Lastly, the input features for the problem should be non-linearly related to the target variable. Most biological phenomena are highly non-linear, so it is rare to encounter a biological problem where input and output are linearly related. This also becomes apparent from examining the AI models that underpin some seminal breakthroughs in the context of biology, such as CellOT for gene perturbation prediction (Bunne et al., 2023), ESMFold and AlphaFold for protein structure prediction (Jumper et al., 2021; Lin et al., 2023b), and EVE and AlphaMissense for missense variant pathogenicity prediction (Frazer et al., 2021; Cheng et al., 2023). To model the non-linearity inherent to these problems, non-linear activation functions are one of the key elements allowing AI models to effectively capture the highly complex relationships within the underlying distributions they attempt to model. Since many biological phenomena exhibit strong non-linearity, it makes sense to express and solve these problems in the language of AI.

## AI methods and data modalities in drug target discovery

One leading reason for the convergence between AI and drug discovery is the diverse range of data types that are being used in drug discovery. The data can be presented in various forms, such as tabular, text, sequences, graphs, and images, each offering a distinct perspective into the biology underlying disease and potential cures. In Table 1, we summarise the different modalities, their use-cases, and some open-access data sources. In the following paragraphs, we briefly discuss each data modality, and how it is generally used in drug target discovery.

**Table 1.** Categorisation of various data modalities commonly used in the field of biomedical research and drug target discovery, along with biology the data represents, the primary AI architecture employed on them, and key data sources

Data modality	Biological representation	Main AI architectures	Example data sources^1^
Tabular	Multiomics, electronic health records	Traditional machine learning,^2^ multilayer perceptron	UK Biobank, Genes & Health, OpenTargets, TCGA, GEO
Text	Gene ontology, scientific literature, clinical trials	Large language model	GO, PubMed, ClinicalTrials.gov
Sequence/structure	DNA, RNA, protein, small molecules	Attention–based neural network, generative model, language model	Ensembl, UniProt, UCSC Genome Browser, ChEMBL, GenBank, PDB, GENCODE
Graph	PPI, gene interaction network, protein structures, small molecule structures, pathway annotations	Graph neural network	STRING, STITCH, BioGRID, PDB, TRRUST, RegNetwork, IntAct, PubChem, ChEMBL, Reactome, KEGG
Image	Histopathology, radiology, spatial transcriptomics	Convolutional neural network, visual transformers	TCIA, GDC, MICA–MIC

Note that the AI architectures are not exclusive to these data modalities. In practice, multiple modalities are combined or sometimes even integrated into each other in an end-to-end fashion.

1 Citations to databases can be found in Supplementary Material S2.

2 In this case, we mean traditional machine learning to encompass linear and logistic regression, support vector machines and tree-boosting models.

One of the most common methods for presenting data related to drug target discovery is through structured tables. Typically, these tabular data structures will contain information describing genes or variants, for example, allele frequency, mutation type, and conservation scores across species. There are different resources and consortia that aggregate and characterise genomic data in tabular form, such as UK Biobank (Sudlow et al., 2015), Genes & Health (Finer et al., 2020), and Open Targets (Ochoa et al., 2021). Traditional machine learning (ML) methods, for example, XGBoost (Chen and Guestrin, 2016), Linear Regression, Logistic Regression (Pedregosa et al., 2011), as well as deep neural networks (LeCun et al., 2015), have been developed and tailored to tabular datasets. Therefore, these models have a track record of delivering outstanding performance when working with tabular data.

Textual data, comprising scientific literature, research articles, patents, clinical trial reports, medical textbooks, chemical databases and electronic health records, represents a valuable resource for drug discovery. The unstructured information in textual documents can provide us with critical insights related to potential drug targets, novel or repurposed drug candidates, and adverse events amongst others. Textual data is typically best analysed using Natural Language Processing (NLP) methods. Recently, large language models (LLMs) have surfaced as the state-of-the-art model type to analyse textual data. LLMs are deep neural networks that combine many different layer types, such as embedding layers, attention layers and linear layers that coalesce to learn semantic information from textual input. Typically, LLMs are pre-trained using self-supervised approaches where a large corpus of text gets tokenised, that is, it gets mapped to numerical vectors representing the words. This corpus is masked at random, and consequently tasked with predicting the next tokens (Radford et al., 2018; Devlin et al., 2019). For task-specific objectives, the pre-trained model can be trained further on data related to the task of interest, for example, information retrieval or translation (Microsoft Research AI4Science and Microsoft Azure Quantum 2023; Singhal et al., 2023a, 2023b).

Data that can be represented sequentially are fundamental to biology. Such sequences often correspond to biological or chemical structures. Some of these data are genomic data, transcriptomic data, protein sequences, and drug compound libraries in the form of SMILES or SELFIES strings. Previously, we introduced language models within the context of natural language. Yet, their versatility transcends the domain of language. Language models also prove adept at understanding biological languages, for example, decoding semantic meaning from DNA via nucleotide sequences, and unravelling structural or functional information for proteins through the interpretation of amino acid sequences. To model and use these sequences, language models can be trained to predict masked nucleotides or amino acids and consequently generalise to unseen sequences (Dee, 2022; Benegas et al., 2023; Lin et al., 2023b). Another type of model showing promise in sequential and structural data are generative models. Generative models are self-supervised machine learning models that are trained to model the statistical distribution of input data, typically by reconstructing the original distribution after random noise has been added as input during the training process (Goodfellow et al., 2014). A couple of ways in which these models can be applied are to model DNA regulatory sequences (Zrimec et al., 2022), and they can be utilised to generate novel protein structures that meet some specified criteria. (Ingraham et al., 2023; Watson et al., 2023). Attention-based neural networks have been shown to be well-versed in analysing sequences to correct consensus sequence errors (Baid et al., 2023), comprehend protein structures (Baek et al., 2021; Lin et al., 2023b), and discover potential drug targets (Chen et al., 2023). The attention mechanism allows the model to learn relations between different parts of the input sequence, even if these parts are located far away from each other in their representation space (Vaswani et al., 2017). The most notable example of an attention-based neural network working with sequence-based data is AlphaFold. AlphaFold predicts protein structure in 3D from an amino acid sequence input (Jumper et al., 2021).

Network data (e.g., gene and protein interaction networks) can provide a comprehensive view of molecular relationships by representing them efficiently as graphs with nodes and edges. Furthermore, representing data as a graph allows us to build Graph Neural Networks (GNNs) (Veličković, 2023). GNNs are optimised to learn and propagate information across nodes, allowing for efficient learning from these data structures. In the context of drug target discovery, there are various successful examples of graphs being used, such as in network expansion for pleiotropy mapping (Barrio-Hernandez et al., 2023), CausalBench (Chevalley et al., 2022), and many others (Muzio et al., 2021). A recent trend in drug target discovery has been the usage of knowledge graphs (KGs). These typically are heterogeneous graphs that store different data about compounds or genes in nodes, and relationships between nodes in the edges (Chandak et al., 2023).

Medical imaging, including X-rays, CT scans, MRI and histopathology slides, function as important assets for disease diagnosis and tracking treatment responses. Generative models, convolutional neural networks (CNNs), visual transformers (ViTs) and deep learning architectures are frequently used for the analysis of visual data (Liu et al., 2017; Dosovitskiy et al., 2021; Tu et al., 2023). When it comes to molecular imaging, images are captured in various resolutions all the way down from the tissue to the cellular level. These images offer profound insights into the molecular intricacies of diseases and drug interactions. Finally, drug screening assays generate a treasure trove of image data, showcasing cells or organisms under perturbation of various compounds in pursuit of potential drugs. AI models help with their ability to comprehensively analyse the resulting images. Next to interpreting the images, using image data also often involves image correction and automatic feature extraction, both tasks in which AI methods excel (Dee et al., 2023; Krentzel et al., 2023).

While it is true that certain data modalities conventionally have been associated with certain types of AI architectures, a lot of the state-of-the-art models do not exclusively use a single data modality or a single architecture. Often, data and model types are combined. This combination can occur in various ways, and often different model types are involved with the processing of various types of data before it gets combined, which often happens in so-called embeddings (Ngiam et al., 2011; Venugopalan et al., 2021; Alwazzan et al., 2023; Khader et al., 2023). Embeddings are representations of the raw input data in a latent space that can be used for downstream computations. Furthermore, most modern-day AI architectures consist of various blocks, which are organisational units in a neural network that are composed of different layers, or even whole models that feed into each other and interact with each other. Models like this are often referred to as multimodal machine learning models.

## Exploring AI-based strategies for drug target identification

The first example we will explore is DrugnomeAI, an ensemble architecture for the prediction of drug targets (Polikar, 2006; Vitsios and Petrovski, 2020; Raies et al., 2022). DrugnomeAI excels in predicting the druggability of candidate drug targets by leveraging 324 gene-level features for every protein-coding gene within the human exome. Raies et al. conducted a feature importance study with Boruta, which is a feature selection technique that helps identify the most relevant variables in a dataset by comparing their importance to that of randomised, noise-added variables (Kursa et al., 2010). This analysis showed that the most informative features for druggability prediction were protein–protein interaction features. This is in line with existing research showing that partners of druggable genes are also likely to be druggable (Finan et al., 2017). Raies et al. frame their model’s objective as a positive-unlabelled learning (PUL) problem. Here, the positive dataset comprises targets for which they have identified evidence of druggability, while the unlabelled set encompasses the remaining targets. The ultimate task is to rank these remaining targets based on their predicted druggability. Within their PUL framework, Raies et al. use eight separate classifiers that are stochastically trained through a 10-fold cross-validation process. Subsequently, the predictions from these classifiers are combined to produce the final ranking of the unlabelled drug targets. Notably, Raies et al. observed that the top-ranked genes in their prioritisation exhibit significant enrichment in the clinical literature, arguing that their model has effectively recognised druggability patterns within the feature set.

It is also possible to combine multiple data modalities in a more direct way than ensemble modelling, namely *via* multitask learning (Caruana, 1998). A multitask learning problem in drug target discovery is typically framed as one where you are trying to predict target qualities as well as properties of the target-binding drug (Sadawi et al., 2019; Lin et al., 2022). Multitask learning allows the model to co-learn a set of tasks together to optimise overall performance. This approach leverages shared information between tasks, combatting overfitting and improving generalisation. Multitask neural networks can integrate data from various sources, making them valuable for a wide range of tasks, such as predicting drug targets, but also drug toxicity and sensitivity (Costello et al., 2014; Ammad-Ud-Din et al., 2017). Furthermore, they offer a means to bridge the gap between biology and chemistry in drug discovery by incorporating structural data like SMILES representations, next to information characterising the biological target, enabling simultaneous prediction of side effects, ligand docking, likely targets and key compound properties (Mikolov et al., 2013b, 2013a).

In some areas of study where data is sparsely available, such as for rare diseases or diseases in clinically unavailable tissues, AI methods can meaningfully identify candidate drug targets through transfer learning. Transfer learning is a concept in AI where we train on abundant data that is tangentially related to some problem with limited data, and consequently fine-tune the resulting model towards the limited data case (Pan and Yang, 2010). One example of a model utilising transfer learning is Geneformer (Theodoris et al., 2023). Geneformer uses self-attention to pick out important genes using transcriptomic data, which can vary across different cell types, developmental stages, or disease conditions. Geneformer was trained with a dataset called Genecorpus-30M, which was assembled from 29.9 million human single-cell transcriptomes. The transcriptome data is processed through six transformer encoder units involving self-attention and feed-forward layers. Pre-training is done using a masked learning objective, where 15% of genes in each transcriptome are masked, and the model learns to predict the masked genes based on the context of the unmasked genes. Due to the size and broad scope of Geneformer’s pre-training, together with the potential to fine-tune the model, we refer to this model as a foundation model (Bommasani et al., 2022). Using Geneformer, cardiomyocytes from three types of limitedly available heart tissue were studied: healthy (*n* = 9), hypertrophic cardiomyopathy (*n* = 11), or dilated cardiomyopathy (*n* = 9). Theodoris et al. performed *in silico* treatment analysis by either inhibiting or activating pathways and seeing if this would move the healthy cell states towards either hypertrophic or dilated cardiomyopathic states. If so, the pathway was inspected for potential therapeutic targets based on druggability and disease relevance. A target that was highlighted through this analysis was *ADCY5*, which is a known druggable target (Wagner et al., 2016a) as well as involved in longevity and protection of cardiomyocytes in mouse models (Ho et al., 2010). Another target that *in silico* treatment analysis pointed to in this context was *SRPK3*, which is a downstream effector of *MEF2* (Nakagawa et al., 2005). *MEF2* is known to play a role in myocardial cell hypertrophy (Akazawa and Komuro, 2003). While single-cell foundation models have demonstrated impressive results in certain situations and seem conceptually attractive for downstream applications, it is important to exercise caution. These pre-trained models may not perform well in all contexts, particularly for zero-shot prediction in other biological contexts (Kedzierska et al., 2023). Therefore, employing biological foundation models for zero-shot prediction in contexts divergent from their original training objective should be approached carefully.

GNNs are also being employed in drug target discovery. One such approach is EMOGI (Schulte-Sasse et al., 2021), a graph convolutional network (GCN) that predicts cancer drug targets. EMOGI stands out by integrating a wide range of interaction and multiomics data to predict cancer genes. This way of combining different data sources addresses the evolving understanding of cancer as a complex interplay of genetic and non-genetic factors (Bell and Gilan, 2020; Hanahan and Weinberg, 2011). Unlike previous approaches that primarily rely on somatic mutations and occasionally integrate PPI networks (Cowen et al., 2017; Leiserson et al., 2015; Reyna et al., 2018), EMOGI employs GCNs to predict cancer genes by amalgamating multiple data modalities, including mutations, copy number variations, DNA methylation, gene expression, and PPI networks. The graph is constructed to have its topology represent a PPI network. This means that the nodes represent genes, and the edges represent whether two genes interact. R. Schulte-Sassen et al. also did an interpretability analysis of their GCN model. They use the layer-wise relevance propagation (LRP) propagation rule (Bach et al., 2015), which allows for dissecting what is happening in the GCNs and provides us with insights into why specific genes are classified as cancer-related. Through biclustering and LRP analysis, distinct modules of newly predicted cancer genes (NPCGs) are revealed—some predominantly influenced by network interactions, others primarily driven by omics features. These NPCGs, while not always necessarily displaying recurrent alterations themselves, interact with known cancer genes, positioning them as significant players in tumorigenesis. Notably, these predictions align with essential genes identified through loss-of-function screens, reinforcing the credibility of EMOGI’s insights.

Beyond academic research and applications, as of Q3 2023, there are a plethora of AI-derived therapeutics in clinical trial pipelines. Most of these come forth out of industrial research laboratories. A lot of the information that is publicly available on how AI is influencing drug target discovery comes from what we here refer to as AI-first drug discovery companies. These are companies that highlight explicitly the fact that they are using AI in their drug target discovery and drug design efforts. While we can only associate drugs being AI-derived from such companies, we should note that big pharmaceutical companies are also heavily investing in introducing AI into their pipelines. However, it is much harder to attribute the involvement of AI in the development of new pharmaceuticals in this case. So, while looking at the status of AI-first companies might be a good probe into the penetrance of AI into the pharmaceutical industry, it does not provide us with a comprehensive view of the role AI is currently playing in the industry.

**Figure 2.**
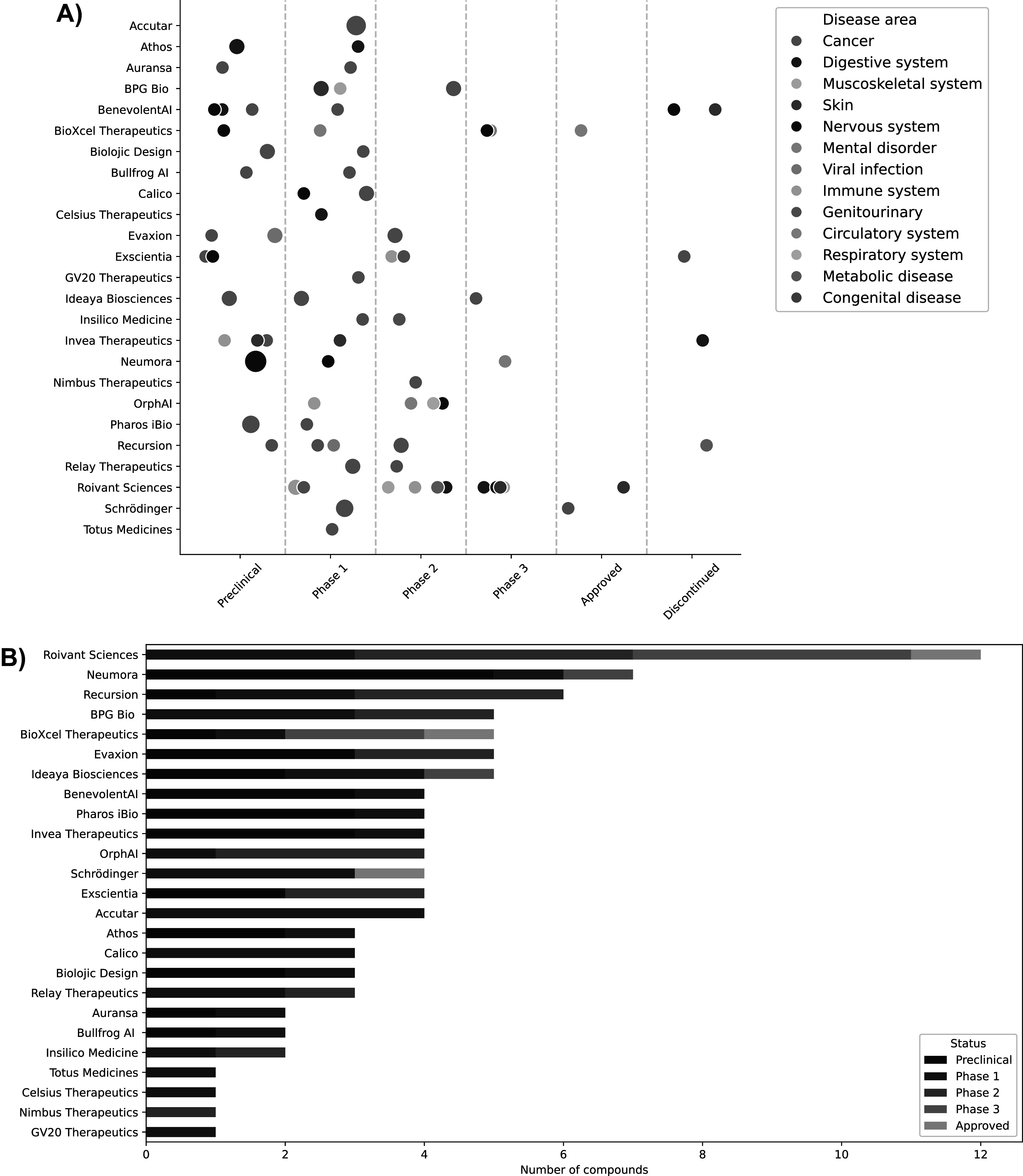
A) Compounds of AI-first companies that are currently in clinical trials, approved or discontinued, stratified by ICD10 disease categories. Scatter size indicates the number of compounds in that clinical trial phase for that company and disease area. Note that dots have been jittered for visual purposes. This does not reflect progress of the compound in the respective phase. B) Number of compounds each company has in clinical trials, where the bar colours refer to the phase or the status of the clinical trial.

In Figure 2, we have visualised the status of targets and associated compounds currently in clinical and preclinical trials. The data was put together by searching and collecting a list of publicly and privately held companies that explicitly mention the usage of AI on their website. We have added a table containing the data we collected in Supplementary Table S1. Note that this is not an exhaustive list, and we only included target-compound pairs for which we could find sufficient data in the pipelines reported by the companies. For discontinued compounds, press-releases and historical website snapshots have been consulted to confirm the development status of compounds. The discontinued compounds collected in our data are an underestimation of the true number of discontinued compounds. Often, data and status on discontinued compounds are not easily accessible in public records. Hence, the only discontinued compounds added to this list are ones that (i) have had accessible press coverage, (ii) have been withdrawn from a clinical trial investigation as indicated by ClinicalTrials.gov, or (iii) have been mentioned in an accessible snapshot of a company’s pipeline webpage, consulted *via* wayback.archive.org, and removed without any mention of success. We only consider compounds in which the company was leading the effort for approval. We use FDA approval status to determine whether a compound has been officially approved. We excluded AI-first companies that have not yet had at least one compound enter clinical trials.

## Discussion and future prospects

AI is penetrating all levels of drug discovery, including target discovery and validation. AI methods rely on the existence of large, high-quality data sets. Currently, these data exist but are certainly incomplete and potentially confounding in nature. We must take note of the limitations of existing data and look at ways to improve data in a targeted manner. Most publicly available big data sets often rely on aggregated information descendent from skewed representations of the population. Different populations display widely varying genomic characteristics and responses to drugs, and consequently, less represented populations suffer from diminished treatment outcomes (Ramamoorthy et al., 2015; Popejoy and Fullerton, 2016; Gross et al., 2022). Therefore, the databases used to identify drug targets often lack sufficient representation of population diversity, resulting in disparate health outcomes for diseases that are effectively treated in well-represented groups but remain challenging to address in underrepresented populations (Hindorff et al., 2018; Landry et al., 2018).

At the molecular level, we encounter a different set of biases in the data we use to train our models. For example, some protein classes are significantly overrepresented compared to others based on FDA approval data, which may be attributed to shared structural or functional similarities for proteins within a given class. If we train a new generation of models with these targets as labels, we are likely to perpetuate these biases in newly prioritised drug targets. Furthermore, we should also acknowledge that because of data availability limitations, bias and historical momentum around known drug targets and classes of targets, there is a significant portion of the genome of which we know too little to assess their validity as drug targets (Finan et al., 2017; Oprea et al., 2018; Wood et al., 2019). Assuming there are also potential drug targets hidden within what has been colloquially termed the “unknome” (Rocha et al., 2023), this would increase the search space of potential drug targets further beyond what the current paradigm of what drug target druggability models consider. Another challenge is that the concept of a druggable target is not static. This is particularly pronounced for cancer, where target-associated pathways are prone to quickly becoming resistant to treatment through various mechanisms (Shabani and Hojjat-Farsangi, 2016). This means that the “one disease, one target” paradigm might not be the best approach to curing diseases, even in cases where a single target is indeed initially therapeutically receptive to treat the disease.

While AI-powered drug target discovery has its fair share of obstacles to overcome, it is still a field that is in its infancy. Moreover, next to these obstacles lie many opportunities for promising discoveries. This is not only limited to drug target discovery, but drug discovery in its broadest sense. For the successful application of AI, specifically deep learning-based architectures, the three guiding principles must be satisfied: scale, complexity and non-linearity. We argue that drug target discovery satisfies all three of these principles. Given this reality, AI-based methods stand to improve the speed with which we can discover and validate novel drug targets. Recent breakthroughs in AI have led to improvements by providing an increased ability to incorporate sequence and structure-based target evidence. As models like AlphaFold are improved and extended to also reflect the dynamic nature of proteins, and we incorporate small molecules and macromolecular structures into these models, our ability to do *in silico* drug discovery will dramatically improve. In addition to predicting protein structures, AI methods stand to significantly improve a multitude of other biological challenges. These include, but are not limited to, predicting gene perturbations, assessing the effects of genetic variants, *de novo* generation of proteins, and molecular docking simulations. In the long run, transitioning a significant portion of the drug discovery pipeline to an *in silico* environment holds substantial advantages for all parties involved with drug discovery. For patients, this shift would enhance the efficiency of developing new and safe medications, resulting in faster delivery of improved therapeutics. For pharmaceutical companies, this transition would lead to significant cost and time savings, which are estimated between 25% and 50% up to the preclinical stage (Loynachan et al., 2023). For us to get to this point, experimental validations of *in silico* methods remain essential both to validate computational predictions and to provide labels for the models to train with.

AI-driven drug target discovery presents a promising avenue for identifying novel, safe and efficacious targets. By leveraging the abundance of multiomics data and the power of modern AI architectures applicable to a variety of data modalities – ranging from images to sequences and protein structures, we find ourselves at the precipice of having data and method converge at meaningful impact on drug target discovery, and drug discovery at large.

## Supporting information

Wenteler et al. supplementary material Wenteler et al. supplementary material

## Supplementary material

The supplementary material for this article can be found at http://doi.org/10.1017/pcm.2024.4.
