# Supplementary material for: AI approaches for the discovery and validation of drug targets
Source: Camb Prism Precis Med. 2024 May 24;2:e7. doi: 10.1017/pcm.2024.4 (PMC11383977; doi:10.1017/pcm.2024.4)
Supplement: Wenteler et al. supplementary material [file S2752614324000048sup001.docx]

**Supplementary Materials**

**S1. Table of AI-First Drug Discovery Companies**

| Company | Target | Disease | Compound | Status |
| --- | --- | --- | --- | --- |
| BenevolentAI | TRK  HRH3  PDE10  Undisclosed  Undisclosed | Atopic Dermatitis  Narcolepsy  Ulcerative Colitis  GM^~~1~~^  ALS^~~1~~^ | BEN-2293  BEN-2001  BEN-8744  BEN-28010  BEN-34712 | Discontinued  Discontinued  Preclinical  Preclinical  Preclinical |
| Relay Therapeutics | PI3Kα  PI3Kα  FGFR2  SHP2 | Breast Cancer  Breast Cancer  Solid Tumors  Solid Tumors | RLY-5836  RLY-2608  RLY-4008  GDC-1971 | Phase 1  Phase 1  Phase 1/2  Phase 1 |
| GV20 Therapeutics | IGSF8 | Solid Tumors | GV20-0251 | Phase 1 |
| Celsius Therapeutics | TREM1 | IBD^~~1~~^ | CEL-383 | Phase 1 |
| Neumora | KOR  V1aR  M4R  NMDA  CK1δ  NLRP3  GCASE | Depression/NPD^~~1~~^  Alzheimer’s/PTSD^~~1~~^  Schizophrenia  Schizophrenia  ALS^~~1~~^/Parkinson’s  Parkinson’s  Parkinson’s | NMRA-140  NMRA-511  NMRA-M4R  NMRA-NMDA  NMRA- CK1δ  NMRA-NLRP3  NMRA-GCASE | Phase 2/3  Phase 1  Preclinical  Preclinical  Preclinical  Preclinical  Preclinical |
| Insilico Medicine | Undisclosed  USP1 | Kidney Fibrosis/IPF^~~1~~^  Breast Cancer | INS-018_055  ISM-3091 | Phase 2  Phase 1 |
| Evaxion | Neoantigen  Neoantigen  Neoantigen NSaA^1~~2~~^  NNgA^1~~2~~^ | Melanoma  Melanoma  Lung cancer  *S. aureus*  *N. gonorrhoeae* | EVX-01  EVX-02  EVX-03  EVX-B1  EVX-B2 | Phase 2  Phase 1/2  Preclinical  Preclinical  Preclinical |
| Athos | Undisclosed  Undisclosed  Undisclosed | IBD^~~1~~^  IBD^~~1~~^  IBD^~~1~~^ | ATH-063  ATH-105  ATH-131 | Phase 1  Preclinical  Preclinical |
| OrphAI | C9ORF72  mTOR  Undisclosed  mTOR | ALS^~~1~~^  PAH^~~1~~^  BO^~~1~~^  PS^~~1~~^ | AIT-101  LAM-001  LAM-001  LAM-001 | Phase 2  Phase 2  Phase 2  Phase 1 |
| Accutar | ER  ER  AR  BTK | Breast Cancer  Breast Cancer  Prostate Cancer  Blood Cancer | AC-0699  AC-0682  AC-0176  AC0676 | Phase 1  Phase 1  Phase 1  Phase 1 |
| Schrödinger | IDH1  MALT1  CDC7  CDC7 | AML^~~1~~^  NHL^~~1~~^  Blood Cancer  Solid Tumor | AG-120  SGR-1505  SGR-2921  SGR-2921 | Approved^~~3~~2^  Phase 1  Phase 1  Phase 1 |
| Exscientia | CDK7  PKCθ  LSD1  MALT1  A_2A_ | Solid Tumor  ID^~~1~~^  AML^~~1~~^  Blood Cancer  Kidney/Lung Cancer | GTAEXS-617  EXS-4318  EXS-74539  EXS-73565  EXS-21546 | Phase 1/2  Phase 1/2  Preclinical  Preclinical  Discontinued |
| Recursion | Undisclosed  NF2  APC  AXIN1  Undisclosed  RBM39  GM2 | CCM^~~1~~^  Brain Cancer  FAP^~~1~~^  Solid Tumor  *C. difficile*  Ovarian Cancer  Gangliosidosis | REC-994  REC-2282  REC-4881  REC-4881  REC-3964  Undisclosed  REC-3599 | Phase 2  Phase 2  Phase 2  Phase 1  Phase 1  Preclinical  Discontinued |
| Totus Medicines | PI3KCA | Solid Tumors | TOS-358 | Phase 1 |
| Calico | TDP43  Undisclosed  Undisclosed | ALS  Solid Tumor  Solid Tumor | ABBV-CLS-7262  ABBV-CLS-484  ABBV-CLS-579 | Phase 1  Phase 1  Phase 1 |
| Biolojic Design | IL2  IL13 / TSLP  IL2 | Solid Tumor  Solid Tumor  Solid Tumor | AU-007  BD-9  BD-3 | Phase 1  Preclinical  Preclinical |
| BPG Bio | Undisclosed  Undisclosed  Undisclosed  Undisclosed  Undisclosed  Undisclosed | Epidermolysis Bullosa  CIA^~~1~~^  Sarcopenia  SCC^~~1~~^  Pancreatic Cancer  GM^~~1~~^ | BPM-31510  BPM-31510  BPM-31510  BPM-31510  BPM-31510  BPM-31510IV-11 | Phase 1  Phase 1  Phase 1  Phase 2  Phase 2  Phase 2 |
| Auransa | Undisclosed  Undisclosed | Liver Cancer  Heart-Safe Chemo | AU-409  AU-018 | Phase 1  Preclinical |
| Invea Therapeutics | CMA1  CMA1  CMA1  CARD5  5HT_2~~3~~_ | Atopic Dermatitis  Chronic Urticaria  ISM^~~1~~^  ID^~~1~~^  IBD^~~1~~^ | INVA-8001  INVA-8001  INVA-8001  INVA-8003  INVA-8002 | Phase 1  Preclinical  Preclinical  Preclinical  Discontinued |
| Bullfrog AI | Undisclosed  Undisclosed | Glioblastoma  Glioblastoma | BF-222  BF-223 | Phase 1  Preclinical |
| Pharos iBio | Undisclosed  Undisclosed  Undisclosed  RAF/DDR | AML^~~1~~^  Ovarian Cancer  Breast Cancer  Solid Tumor | PHI-101  PHI-101  PHI-101  PHI-501 | Phase 1  Preclinical  Preclinical  Preclinical |
| Nimbus Therapeutics | HPK1 | Solid Tumor | NDI-101150 | Phase 1/2 |
| BioXcel Therapeutics | Alpha-2  Alpha-2  Alpha-2  Alpha-2  Undisclosed | Schizophrenia/BPD^~~1~~^  Alzheimer’s  Schizophrenia/BPD  Depression  Alzheimer’s | Dexmedetomidine  BXCL-501  BXCL-501  BXCL-501  BXCL-502 | Approved  Phase 3  Phase 3  Phase 1  Preclinical |
| Ideaya Biosciences | PKC  MAT2A  PARG  POLQ  WRN | Uveal Melanoma  Solid Tumor  Breast/Ovarian Cancer  Solid Tumor  Gastrointestinal Cancer | Darovartesib  IDE-397  IDE-161  GSK-101  WRN | Phase 2/3  Phase 1  Phase 1  Preclinical  Preclinical |
| Roivant Sciences | AhR  AhR  TL1A  TL1A  FcRn  FcRn  FcRn  FcRn  TYK2/JAK1  TYK2/JAK1  CSF2  SF3B1 | Psoriasis  Atopic Dermatitis  Ulcerative Colitis  Crohn’s Disease  Myasthenia Gravis  Thyroid Eye Disease  WAIHA^~~1~~^  Autoimmune Disease  Dermatomyositis  SLE^~~1~~^  Sarcoidosis  MDS | Tapinarof  Tapinarof  RVT-3101  RVT-3101  Batoclimab  Batoclimab  Batoclimab  IMVT-1402  Brepocitinib  Brepocitinib  Namilumab  RVT-2001 | Approved  Phase 3  Phase 3  Phase 2  Phase 3  Phase 2  Phase 1  Phase 1  Phase 3  Phase 2  Phase 2  Phase 1 |

Table S1: Targets identified and under development in AI-first biopharmaceutical companies. Targets and compound identifiers are being queried from company pipelines and ClinicalTrials.gov.

^~~1~~^GM = Glioblastoma Multiforme; ALS = Amyotrophic lateral sclerosis; IBD = Inflammatory Bowel Disease; NPD Neuropsychiatric Disorders; PTSD = Post-Traumatic Stress Disorder; IPF = Idiopathic Pulmonary Fibrosis; PAH = Pulmonary Arterial Hypertension; BO = Bronchioliti~~t~~s Obliterans; PS = Pulmonary Sarcoidosis; AML = Acute Myeloid Leukemia; NHL = Non-Hodgkin Lymphoma; ID = Inflammatory Disease; CCM = Cerebral Cavernous Malformation; FAP = Familial Adenomatous Polyposis; CIA = Chemotherapy-induced Alopecia; SCC = Squamous Cell Carcinoma; ISM = Indolent Systemic Mastocytosis ; CN = Cutaneous Neurofibroma; BPD = Bipolar Disorder; WAIHA = Warm Autoimmune Hemolytic Anemia; SLE = Systemic Lupus Erythematosus; MDS = Myelodysplastic Syndromes.

^1~~2~~^NSaA = Novel *S. aureus* Antigens; NNgA = Novel *N. gonorrhoeae* Antigens

^2~~3~~^In collaboration with Agios Pharmaceuticals. Acquired by Servier.

**S2. Database Citations**

In order of appearance in Table 1: UK Biobank (Bycroft *et al.* 2018), Genes & Health (Finer *et al.* 2020), OpenTargets (Ochoa *et al.* 2021), TCGA (Weinstein *et al.* 2013), GEO (Clough and Barrett 2016), GO (Ashburner *et al.* 2000), Ensembl (Martin *et al.* 2023), UniProt (The UniProt Consortium 2023), UCSC Genome Browser (Kent *et al.* 2002), ChEMBL (Mendez *et al.* 2019), GenBank (Sayers *et al.* 2021), PDB (Berman *et al.* 2000), GENCODE (Frankish *et al.* 2021)

STRING (Szklarczyk *et al.* 2023), STITCH (Kuhn *et al.* 2008), BioGRID (Stark *et al.* 2006), TRRUST (Han *et al.* 2018), RegNetwork (Liu *et al.* 2015), IntAct (Kerrien *et al.* 2007), Reactome (Gillespie *et al.* 2022), KEGG (Kanehisa *et al.* 2023), TCIA (Clark *et al.* 2013), GDC (Grossman *et al.* 2016),MICA-MIC (Royer *et al.* 2022)
