# Supplementary material for: AI approaches for the discovery and validation of drug targets
Source: Camb Prism Precis Med. 2024 May 24;2:e7. doi: 10.1017/pcm.2024.4 (PMC11383977; doi:10.1017/pcm.2024.4)
Supplement: Peer review history

# Editor report: Recommendation: AI approaches for the discovery and validation of drug targets — R0/PR2

**DOI:** 10.1017/pcm.2024.4.pr2

**Affiliations:** Canon Medical Research Europe Ltd, United Kingdom of Great Britain and Northern Ireland

This is an excellent, well written and highly relevant paper that is suitable for publication with some minor revisions.

# Editor report: Decision: AI approaches for the discovery and validation of drug targets — R0/PR3

**DOI:** 10.1017/pcm.2024.4.pr3

**Affiliations:** Institute of Cardiovascular & Medical Sciences, University of Glasgow College of Medical Veterinary and Life Sciences, United Kingdom of Great Britain and Northern Ireland

No accompanying comment.

# Editor report: Recommendation: AI approaches for the discovery and validation of drug targets — R1/PR5

**DOI:** 10.1017/pcm.2024.4.pr5

**Affiliations:** Canon Medical Research Europe Ltd, United Kingdom of Great Britain and Northern Ireland

No accompanying comment.

# Editor report: Decision: AI approaches for the discovery and validation of drug targets — R1/PR6

**DOI:** 10.1017/pcm.2024.4.pr6

**Affiliations:** Institute of Cardiovascular & Medical Sciences, University of Glasgow College of Medical Veterinary and Life Sciences, United Kingdom of Great Britain and Northern Ireland

No accompanying comment.
